# BBMRI-ERIC as a resource for pharmaceutical and life science industries: the development of biobank-based Expert Centres

**DOI:** 10.1038/ejhg.2014.235

**Published:** 2014-11-19

**Authors:** Gert-Jan B van Ommen, Outi Törnwall, Christian Bréchot, Georges Dagher, Joakim Galli, Kristian Hveem, Ulf Landegren, Claudio Luchinat, Andres Metspalu, Cecilia Nilsson, Ove V Solesvik, Markus Perola, Jan-Eric Litton, Kurt Zatloukal

**Affiliations:** 1 Department of Human Genetics, Leiden University Medical Center, Leiden, The Netherlands; 2 Institute for Molecular Medicine Finland, University of Helsinki, Helsinki, Finland; 3 Institut Pasteur, Paris, France; 4 BIOBANQUES Infrastructure, Inserm US 13, Hopital de la Salpetriere, Paris, France; 5 Department of Immunology, Genetics and Pathology, Science for Life Laboratory, Uppsala University, Uppsala, Sweden; 6 Department of Public Health and General Practice, Norwegian University of Science and Technology, Trondheim, Norway; 7 Magnetic Resonance Center, University of Florence, Florence, Italy; 8 The Estonian Genome Center, University of Tartu, Tartu, Estonia; 9 Uppsala University Innovation, Science for Life Laboratory, Uppsala University, Uppsala, Sweden; 10 Lifandis AS, Levanger, Norway; 11 Department of Chronic Disease Prevention, National Institute for Health and Welfare, Helsinki, Finland; 12 Biobanking and Biomolecular Resources Research Infrastructure-European Research Infrastructure Consortium (BBMRI-ERIC), Graz, Austria; 13 Institute of Pathology, Medical University of Graz, Graz, Austria

## Abstract

Biological resources (cells, tissues, bodily fluids or biomolecules) are considered essential raw material for the advancement of health-related biotechnology, for research and development in life sciences, and for ultimately improving human health. Stored in local biobanks, access to the human biological samples and related medical data for transnational research is often limited, in particular for the international life science industry. The recently established pan-European Biobanking and BioMolecular resources Research Infrastructure-European Research Infrastructure Consortium (BBMRI-ERIC) aims to improve accessibility and interoperability between academic and industrial parties to benefit personalized medicine, disease prevention to promote development of new diagnostics, devices and medicines. BBMRI-ERIC is developing the concept of Expert Centre as public–private partnerships in the precompetitive, not-for-profit field to provide a new structure to perform research projects that would face difficulties under currently established models of academic–industry collaboration. By definition, Expert Centres are key intermediaries between public and private sectors performing the analysis of biological samples under internationally standardized conditions. This paper presents the rationale behind the Expert Centres and illustrates the novel concept with model examples.

## BBMRI, the European Biobank Infrastructure

Most of our current knowledge of diseases, as well as available diagnostic assays and drugs, were obtained through systematic investigation of human biological samples and medical data. A key to such investigations is the facilitation of structured access to quality-controlled patient–donor samples and data in an ethically appropriate and privacy-compliant manner. For decades, human biological samples and data have been gathered in the so-called biobanks, that is, collections of biological samples with connected information about the donors and results of prior analyses.

In the recent decade, several international initiatives have emerged to coordinate and harmonize biobanking, and to develop regionally and ultimately globally, standardized protocols and metrics. A main characteristic of these initiatives, distinguishing them from specific, hypothesis-driven biomedical research projects, is their *infrastructural* nature, with the aim to facilitate biomedical research by improving generic interoperability. An early and well-known international example is the Canadian-led, Public Population Project on Genomics and Society (P^3^G).^1^

In Europe, the Biobanking and BioMolecular resource Research Infrastructure (BBMRI)^2^ was prioritized in the roadmap of the European Strategy Forum on Research Infrastructures (ESFRI). From 2008 to 2011, BBMRI received preparatory phase funding from the European Framework Programme 7. As of 2009, several European member states initiated linked national BBMRI activities, including BBMRI-NL in the Netherlands and BBMRI.se in Sweden, and today many member states have a ‘BBMRI-xx' programme in or close to operation.^3^

## The launch of BBMRI-ERIC

On 3 December 2013, BBMRI was officially awarded the Community legal framework for a European Research Infrastructure Consortium (ERIC).^4^ BBMRI-ERIC is one of the first infrastructures that emerged from the ESFRI roadmap, which addresses all scientific disciplines that require a large-scale Research Infrastructure with a joint effort on the European or international scale.^5^ In some cases, ‘single-sited' Research Infrastructures provide the best solution for the necessary research. In other cases, a ‘distributed' Research Infrastructure is best suited from the scientific viewpoint as well as for the sustainability and optimization of partially existing resources. BBMRI-ERIC is a distributed infrastructure. This specific legal form is designed to facilitate the joint establishment and operation of research infrastructures of European interest. The ERIC status^4^ allows pulling together biobanks and biomolecular resources into a pan-European facility. BBMRI-ERIC will provide access to the collections of partner biobanks and biomolecular resources, their expertise and services on a non-profit basis.

Founding members of BBMRI-ERIC are Austria, Belgium, Czech Republic, Estonia, France, Germany, Finland, Greece, Italy, Malta, the Netherlands and Sweden, and more states are expected to join in the coming years. Observers are Switzerland, Norway, Poland, Turkey and IARC/WHO (The World Health Organization's International Agency for Research on Cancer). The Office is based in Graz, Austria with Prof. Jan-Eric Litton as its Director General. BBMRI-ERIC also creates a platform for the involved researchers to communicate with policymakers in the EU and the Member States.

## BBMRI-ERIC as a key resource for the life science industry

**Figure 1:**
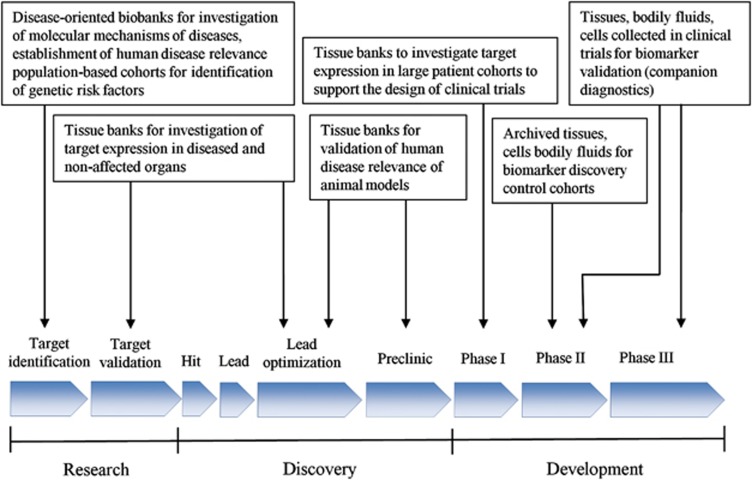
BBMRI-ERIC associated biobanks offer a wide and versatile range of biospecimens (bodily fluids, tissue and cells), covering the whole drug discovery and development process.

The whole process from the early research stage up to preclinical research and further clinical development may be covered with samples provided through BBMRI-ERIC^6^ (Figure 1). Thus, BBMRI-ERIC is set up to become a relevant and important source for partners in academic and scientific institutions as well as in the pharmaceutical and life science industries, thereby contributing directly to the Innovation Union strategic goals. Companies working with biospecimens can be categorized into the three sectors of pharma, diagnostic and biotech industries. BBMRI-ERIC-related markets show significant growth for the global market of biomarkers, with an overall compound annual growth rate (CAGR) of 18%, as foreseen in 2007 for 2007–2012 (Table 1). This 2007 biomarker market prognosis for 2012 was already exceeded in 2010 (Table 2),^7^ and the largest growth occurs in the genomics subsegment, with an expected CAGR of 26.9% for 2010–2015.

### Biotechnology industry market

The number of new biotech firms has increased in all industrial nations over the past two decades. The application areas of biotechnology are quite diverse and include therapeutics, diagnostics, devices and chemicals in the life science sector, as well as in sectors such as agriculture, food and cosmetics and the environmental and energy sector. Most biotechnology applications are relatively new and rapidly evolving. There is no single group of activities that clearly characterizes the biotechnology industry. One possible alternative is to think about the biotechnology industry in terms of the sectors from which those organizations come that are involved in the overall value-added process. Three segments can be distinguished:

- Universities and research institutes where the underlying bioscience is generated, upon which new technologies are often created.
- Dedicated biotechnology firms that rely on the science base and develop new technological procedures and techniques. This group of companies is dominated by small and medium enterprises (SMEs), which are often started as academic spin-offs.
- Biotechnology commercializing firms that apply the technological procedures to application areas.

BBMRI-ERIC has close links to all three segments, all of which have important roles for technology transfer activities to be implemented by BBMRI-ERIC.

### Pharma and biomarker market

The global market for cancer profiling technologies reached US$26.1 billion (€20.3 B) in 2012. This market is expected to grow to nearly US$30.1 billion (€22.7 B) in 2013 and US$54.8 (€41.0 B) billion in 2018, with a CAGR of 12.8% over the 5-year period (2013–2018).^8^ The global biomarker market is estimated to be US$20.5 billion (€15.3 B) by 2014, growing at a CAGR of 19.7% from 2009 to 2014, driven by the high demand for biomarkers in the field of drug discovery.^9^ Genomics has been and will continue to be the fastest-growing driver of biomarker technology,^9^ and is entering even into the food industry.^10^ Pharmaceuticals currently on the market target fewer than 500 human gene products. Even though not all of the 20 000 or so human protein coding genes will have products targetable for drug development, this nonetheless suggests that there is an enormous untapped pool of human gene-based targets for therapeutic intervention.

### Diagnostic industries market

Molecular diagnostics, a new discipline exploiting ‘-omics' technologies to classify and understand diseases, and to assist individuals at particularly high risk, is currently one of the fastest growing segments in the health-care industry. This market is being driven by several growth factors, which include greatly improved technologies for sample analysis and data evaluation. The global market size is expected to expand from US$3.67 billion (€2.78 B) in 2010 to US$6.35 billion (€4.75 B) by the year 2015.^11^

### Biobanking-related market

Biobank consumer and supplier companies generate a significant market by themselves and support regional business development. BBMRI-ERIC creates an incentive for companies to locate in the vicinity of the BBMRI-ERIC partner biobanks and Expert Centres (*vide infra*). Much important expertise and knowledge in applied sciences is by nature not codified but tacit and can only be transferred by direct contact between individuals concerned. Physical proximity supports knowledge and technology transfer as demonstrated by many successful biotech clusters around the world. The biobanking-related market includes companies in the fields of cryotechnology, reagents, plastic ware (eg, cryo tubes, vials and cell culture flasks), robotic sample processing systems, reagents and equipment for sample preanalytics, sample tracking, data management, biosafety and biosecurity, and, importantly, the many different analytical platforms and their attendant data analysis.

The area of bioinformatics is especially important to biobanking, because bioinformatics-based tools are needed to link biospecimens with databases, to analyse data, to set up searchable catalogues and to exchange results. In 2007, the global bioinformatics market was valued at about €1.1 billion. In 2011, its value was estimated at nearly US$2.8 billion (€2.0 B), close to US$3.2 billion (€2.5 B) in 2012, and it is forecasted to grow to nearly $7.5 billion (€5.6 B) by 2017, increasing at a CAGR of 18.7%.^12^ This notably reflects the explosive growth of bioinformatics in pharmacogenomics. The growth of the bioinformatics industry can, according to experts, be attributed to its increased use in the pharmaceutical industry.^13, 14^ The application of bioinformatics in drug discovery and development may reduce both the cost of developing new drugs and their time to market by as much as 30%.^15, 16, 17, 18^ Finally, the growing field of personalized medicine will depend very much on biobanks, and as personalized medicine is getting ready for the mainstream health care and large parts of populations will be included into different biobanks, the BBMRI Expert Centres are well placed to lead the way.

## BBMRI Expert Centres: the rationale

**Figure 2:**
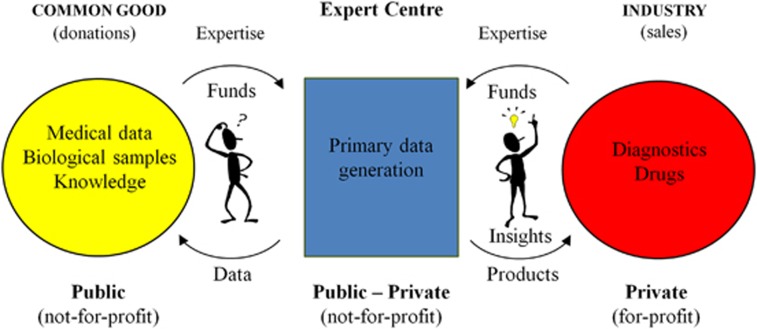
Expert Centre as the key actor providing a trusted, operational middle ground between the public and private sectors.

Cutting edge research as well as further innovations for the life science industry will strongly depend on transnational access for academia and industry to high-quality human biological samples and associated medical information in an efficient and secure manner. The finite key resource of human biological samples is subject to a series of ethical and legal restrictions, thus requiring innovative solutions for efficient utilization. By performing the primary analysis of biological samples under internationally standardized conditions in a precompetitive environment, two major goals are addressed: (1) access to primary data is provided in a form that can more easily be shared than the biological samples themselves, and (2) high quality and over time increasing amounts of information from biological samples is made available to industry for further product development. This should be achieved by ‘BBMRI Expert Centres' associated with BBMRI-ERIC (Figure 2).

BBMRI-ERIC-associated Expert Centres can be non-profit organizations that represent a novel public–private partnership model. They are responsible for the analysis of samples in the country of origin under internationally standardized conditions and the generation of primary data. BBMRI-ERIC-associated Expert Centres integrate precompetitive public and private research and development (R&D) activities by facilitating integrated access not only to biological samples (made available through BBMRI-ERIC) and medical data but also to a broad spectrum of medical and scientific expertise related to the samples, the data and their analysis, thereby extending the offering of biobanks towards standardize sample analysis and integration of academic and industrial expertise.

Thus, a win-w*in situation* is created for both parties by

- enhancing collaborative research,
- using limited resources more efficiently,
- sharing data, technologies, knowledge and expertise,
- ensuring the right code of conduct in dealing with ethical and legal issues,
- reducing complications concerning ownership of samples and primary data and
- increasing competitiveness in academia as well as on the marketplace through product innovation and increased efficacy of R&D.

### Medical expertise

It is becoming more and more important for any investigation to take into account the whole spectrum of medical, scientific and technological aspects related to a given disease. Important issues include what features of a disease may be manifested in a biological sample, and also the clinical correlates of the disease. The entire knowledge base about the disease in question and deep information about the donors are needed to properly interpret the results of an analysis of biological samples. Expert Centres will provide a framework to facilitate the linking of this knowledge to biological samples.

### Technological/analytical expertise and efficacy

The tremendous progress in development of new analytical technologies results in specific sample quality requirements to readily exploit the potential of recent technologies. The very basic problems in the central substrate of research, the samples, may lead to flawed conclusions in the field of biomedical science.^19^ Accordingly, specific know-how about the quality of biomolecules in a sample is important to guarantee proper interpretation of the analytical data generated. This is all the more important as insufficient evidence-based studies are available to predict the impact of variation in sample quality on the data generated by various analytical platforms.

Another consequence of the rapid technological advances is that technology platforms become more and more specialized (eg, most genome centres have established three or more next-generation sequencing (NGS) technologies). Major upgrades have to be implemented approximately every 6 months, resulting in considerable expenses, and the need to continuously implement new procedures. Furthermore, the capacities of these new technologies exceed the requirements of most research groups or even whole universities or companies, particularly SMEs. This has prompted the creation of specialized genome centres that provide research services. Such centres, which resemble in several features the Expert Centres, have the critical mass to quickly adopt new research techniques and statistical tools. New further improved NGS approaches are regularly announced, which will markedly change the sequencing landscape.^20^ Similar developments can be anticipated for protein analyses, where future comprehensive, highly specific and sensitive investigations of the protein composition of biobank samples promise to offer new insights into disease mechanism and to identify valuable targets for diagnostics and drug therapy. It is an important function of some of the centres also to pioneer new investigative approaches at the level of molecular techniques, instrumentation or bioinformatics to provide users new and unique perspectives on biology.

### Expert Centres address ethical and legal restrictions

BBMRI Expert Centres function as a focal point of contact between the public and the private sectors. Human biological samples and medical data are often provided as donations (opt-in donations) or as residual material, and the motivation of their provision is seen as to contribute to the common good (Figure 2). To develop innovative products and maintain or gain market leadership, the private sector not only needs access to biospecimens and data but also the expertise with which these have been gathered to properly interpret results; commercialization of human bodily materials is forbidden according to the Council of Europe's Oviedo Convention^21^ and by national legislation in most Member States, and financial compensation, even on a cost-recovery basis, is generally not accepted by the public. Therefore, only research collaboration can provide a sound basis for accessing human biological samples and associated medical data. This situation may be a source of conflict that makes access for industry difficult or even impossible in some cases.^22^ Several studies also show public reservations for commercial partnership in public-funded research, because of concerns regarding benefit sharing and potential impact on the research agenda. Expert Centres operating on a not-for-profit basis, and with an explicit mission to transparently inform participants on uses of their data and materials, would present a good solution for this problem. The participants' and public engagement is a key element of building the necessary trust.^23^ This will be further developed below.

### Expert Centres as the future ‘highways' for transnational research collaborations

**Figure 3:**
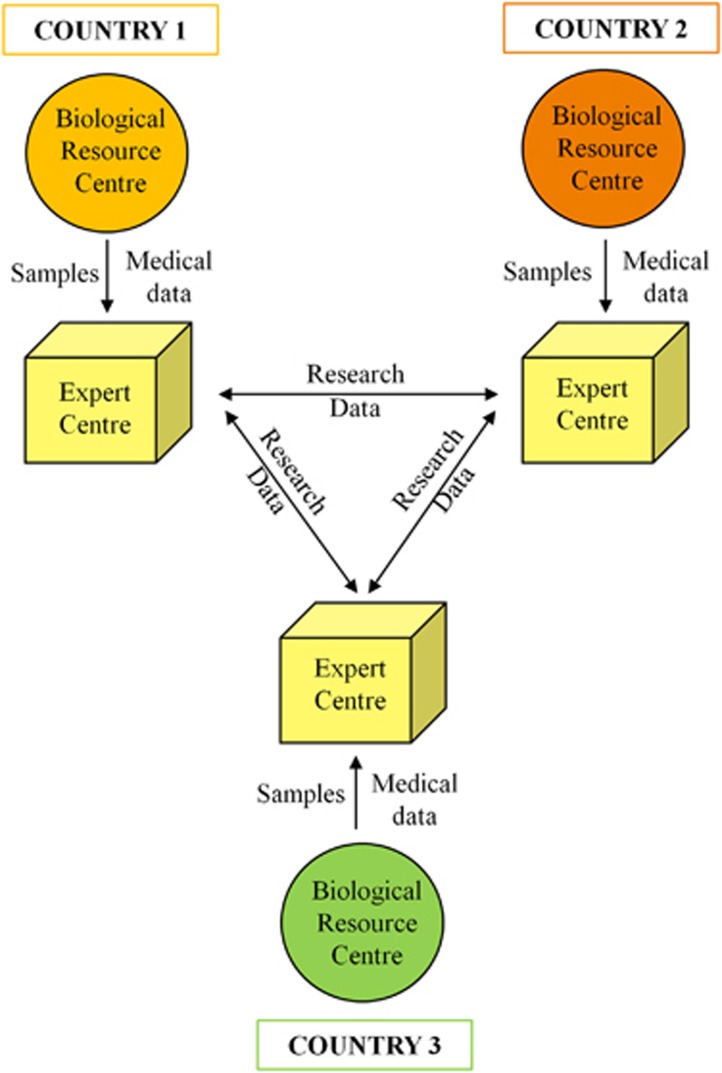
Expert Centres as new ‘highways' for transnational research. In addition to public–private partnerships, the Expert Centre model can be used in public–public collaborations to provide efficient and secure access to samples and data between the collaborative parties.

Several countries, including China, Russia, Brazil and India, have legal restrictions on export of biological samples that make transnational research collaboration difficult.^24^ The establishment of partner Expert Centres in Europe and also non-European countries that operate under the same standards and quality management schemes could generate novel ‘highways' for future transnational research collaborations as samples will be analysed in the country of origin and only research data are shared (Figure 3). This would also guarantee that the country of origin participates in the improvement of health benefit and creation of economic value from public resources. The establishment of a worldwide network of Expert Centres in the context of biobanks and biological resource centres is well in line with the goal of OECD (Organization for Economic Co-operation and Development) to establish a Global Biological Resources Centres Network (GBRCN) to provide efficient and secure access to biological samples as key resources for the advancement of biotechnology and medicine. Thus, Expert Centres can be established as public–private partnerships (Figure 2), and also as public–public collaborations (Figure 3).

### Key features of expert centres

High-quality Expert Centres are characterized by a broad spectrum of medical and scientific expertise, with an ability to provide access to the latest and sometimes unique technologies, adequate IT solutions, exhibiting cost efficacy, high level of standardization, professional quality management, flexible solutions for the generation of intellectual property, confidentiality, ethical and legal compliance, and professional project and partnership management.

### Latest technologies

For optimal biospecimen analysis, a wide spectrum of ‘-omics' analysis platforms needs to be established. It is becoming increasingly challenging, both technically and financially, to remain up to date with the latest developments of analytic procedures. Furthermore, the tremendously increased volume of analytical data and the necessity to correlate this with clinical and scientific information require the development of new IT solutions and statistical tools that will be provided by Expert Centres. The development and implementation of powerful bioinformatics and biostatistics has been arguably a main driver of recent biobanking successes not only in the discovery of risk factors for common diseases but also in developing ‘systems medicine': connecting multiple levels of -omics data and thereby enabling discovery of new biological pathways, disease mechanisms and medications.

### Cost efficacy

The enormous advancements in analysis technologies increasingly make the case for specialized centres as keeping up to date with this development is neither feasible nor affordable for smaller, non-specialized institutions. These developments are in line with the general tendency of outsourcing specific tasks to specialized service providers. Furthermore, the new technologies, particularly in sequencing,^20^ have enormous analytical capacity that cannot be properly used by individual institutions. Therefore Expert Centres can operate at lower costs than classical research laboratories in academia and industry.

### High level of standardization

A major limitation in multicentre studies is that most of the latest ‘-omics' analysis platforms and the related preanalytical processes are not sufficiently standardized to allow proper data integration of analysis performed in different centres. This can be improved by building a network of interacting Expert Centres and implementing harmonized standard operating procedures (SOPs), common certification and accreditation procedures, use of common reference materials and regular participation in ring trials. Data generated by such internationally harmonized and standardized analysis platforms can more readily be combined and integrated in broad, collaborative analyses of biological and medical questions. This results in a more efficient use of the biological materials and generates important added value for the scientific community.

### Professional quality management

Quality management is essential for any industrial R&D, and it becomes increasingly important for academic research as well, especially in joint projects with the industry. Quality management within Expert Centres should take advantage of experiences established in the industry. Appropriate quality management of Expert Centres can be achieved through guidance or supervision by advisory boards that include representatives from the industry.

### Confidentiality

Any project performed for the industry would conform to strict confidentiality regulations, guaranteeing that no company-specific confidential information is disclosed to any other industrial partner or distributed within the academic community.

### Intellectual property

The provision of biological samples or performing analysis using established techniques cannot by itself be considered an inventive step, and therefore does not in principle justify claims for intellectual property by the Expert Centres or a biobank. However, in the context of scientific collaborations between academic and industrial partners, joint intellectual property might be generated by industrial partners and Expert Centres. The exploitation of such intellectual property should be as flexible as possible and could be negotiated between the partners on a case by case basis. The general policy should be that the parties agree in advance how to share the value of any joint intellectual property with the aim to minimize obstacles to exploiting the findings. In case of joint inventions, the academic partners should benefit on the basis of royalties or other benefit sharing models that properly consider the contribution of public resources, expertise and work, whereas industrial partners should have optimal freedom to further develop the intellectual property to successful products. When the Expert Centre generates promising intellectual property without an industrial coinventor, the ambition should still be able to translate the results for industrial and/or medical applications, either by licensing the findings to an industrial partner or by spinning out start-up companies.

### Ethical and regulatory issues

If any field is well aware of the importance of respecting privacy and adhering to ethically acceptable research standards, it is arguably the biobanking community. Biobanking originated in the past by collecting clinical samples for aetiological studies. In clinical biobanking, the particular vulnerability of patients has been a strongly debated ethical issue. Consequently, extensive legislation is in place in all countries on data privacy, the consent status of research samples and the reuse of left-over material, and these matters are regularly revisited in the light of advancing insights and technologies. A more recent development in biobanking is the value of large, prospective cohorts, both to understand epidemiological aspects and to get a better handle on prognosis, early diagnosis and prevention. Here, typically, unaffected participants biobanks are donating their samples and information for the common good (Figure 2) and mostly without expecting a direct health benefit in return. Respecting privacy and confidentiality is essential for both clinical and prospective biobank participants. While in the former, patients are more vulnerable in terms of dependence of health providers, in the latter, participants are more vulnerable given the potential of unforeseen results. A fine, and not always distinguishable line separates protecting participants from unsolicited findings without actionable clinical (or psychological) benefit, and making them aware of putative risks upon which action may be taken. In both types of biobanks, the key issue is to build and preserve trust between the biobanking community on one hand and patients and participants, and also the citizenry, on the other hand.^23^ Infringements on expectations and agreements with respect to sharing of data will greatly damage this trust.

As argued in earlier sections, in the future academic–industry collaboration will have a central role to translate biobank data into actionable solutions. Neither of the parties will be able to do this on its own, as they separately are lacking the necessary combination of resources, expertise and biological materials, and, perhaps even more important, the broad support of the research subjects.

The way forward in the near future should be to enlist the participation of patients and participants in a much more active, bidirectional way than typically has been possible before the current era of fast and secure internet and social media. Under BBMRI-ERIC, several national BBMRI hubs are now establishing public and patient participatory mechanisms and developing online ‘My biobank' interfaces. Here, donors can compare their own provided data with the biobanks' aggregated data in real-time, in a protected environment, and furthermore they could be kept posted on results of studies involving their sample and data. With this in mind, BBMRI-ERIC is developing as one of its first activities, pan-European Common Services in the fields of Ethics and ICT, and it has in its statutes that research involving BBMRI-ERIC biobanks – and by proxy research in BBMRI Expert Centres – must comply with the rules and procedures of the participating biobanks and European-wide accepted ELSI standards.

It is thus of paramount importance that regulations and procedures designed to protect citizens and legally safeguard the trust of the public do not unintentionally hamper or prevent biomedical research that is arguably in the citizens interests.^25^ A case in point is the current concern in the biomedical research community for the planned EU data-protection legislation based on the draft of the European Commission of 25 January 2012, and the recommendations of the Committee on Civil Liberties, Justice and Home Affairs (LIBE) of January and October 2013; a draft law was adopted by the European Parliament on 12 March 2014, which is widely seen, if enacted, to hamper biobanking and biomedical research well beyond the current legally supported principles of sound and ethically responsible practise.^26, 27^

### Implementation

BBMRI Expert Centres will be established outside of BBMRI-ERIC *per se* in Member countries and have to comply with the following key criteria:

- Involvement of research leaders in implementing cutting-edge technologies.
- Application of common quality management systems in cooperation with other Expert Centres with similar focus.
- Use of common standards and reference samples.
- Participation in proficiency testing/ring trials.
- Publication of general SOPs for sample preanalytics, molecular analysis and data processing.
- Establishment of confidentiality and intellectual property (IP) rules.
- Compliance with ethical and legal rules.
- Commitment for efficient handling of contracts and projects.
- Certification (eg, ISO).
- Accreditation by BBMRI-ERIC, with periodic external audits overseen by BBMRI-ERIC management.
- Cooperation agreement between BBMRI-ERIC and Expert Centres that refers to the criteria above.

One of the first activities in establishing Expert Centres, after the delineation of working field and agreement on standard operation protocols, is the provision of reference samples, followed by participation in proficiency testing. The results of proficiency test will then guide the strategy for improved interoperability and further standardization. The implementation plan foresees feasibility demonstration in pilot studies before major financial commitments can be expected. Some pilot studies are already ongoing. Full implementation should be cofinanced from the public and private sectors, and by using funding instruments provided by the European Investment Bank. Major companies have already expressed their interest in BBMRI-ERIC-associated Expert Centres through their participation in the BBMRI Stakeholder Forum and other planning activities during the BBMRI preparatory phase in 2008–2011 (Table 3).

### Models and examples

Given the novelty of the BBMRI Expert Centre concept, for guidance, several models of putative Expert Centres are given below:

- Large-scale (eg, regional or national) technology centres, providing standardized and validated genomics, proteomics or metabolomics services, open to public institutions including biobanks, and to private companies, provided that they commit to international certification procedures and proficiency tests.
- Single large biobanks or consortia of smaller biobanks that commit to provide standardized access to samples and validated analytical procedures in public or public–private settings.
- Combinations of public and private parties that undertake to generate standardized biomedical data to be made available to a wider public community under appropriate, transparent access rules to safeguard ethical and privacy conditions.
- (Bio)Informatics and/or statistical centres offering standardized and validated analytical pipelines to public and private third parties.

### Examples

As examples, several pilot Expert Centres currently under development are given below:

- *EXCEMET (an Expert Centre for metabolomics)*: The goal of EXCEMET is to further develop technologies and standardize procedures for broad application of metabolomics in biological and medical research, as well as in medical diagnostics. EXCEMET will be established as a not-for-profit public–private partnership based on a consortium agreement between participants from academia and industry. The work will include the further development and refinement of harmonized SOPs and the establishment of a common quality assurance system. Technical developments focus on precompetitive issues, such as decreasing minimal sample volume, improvement of sample stabilization and assay sensitivity, reduction of experiment time, data analysis and reduction of costs. EXCEMET will provide guidance to biobanks on sample management for metabolomics. Furthermore, a monodimensional ^1^H NMR spectrum could become part of the metadata associated with each sample stored in biobanks. This information can be used to document sample identity and quality, and might be considered as a key step towards a systematic transformation of biological samples into data that can be shared with a large community of scientists. EXCEMET is a distributed expert centre of leading institutions in different countries in the field of metabolomics. EXCEMET is led by Prof Claudio Luchinat at the Centro Risonanze Magnetiche –CERM/CIRMMP, University of Florence. The consortium agreement between the founding members (Univ. Florence, Medical Univ. Graz, Metanomics Health, Univ. Gothenburg and Univ. Birmingham) has been signed. Several further institutions are in the process of signing.
- *SciLifeLab (Science for Life Laboratory):* SciLifeLab^28^ in Sweden is a recently established Expert Centre, providing access to a broad range of large-scale molecular analyses, state-of-the-art as well as unique technologies and resources developed by the partners, and with bioinformatic support to assist in the interpretation of results. The services are available for both academic and industrial users, and the centre has a strong ambition to also develop new technologies and reagent resources. Connected to SciLifeLab are scientists with large biobanks, and with advanced expertise in a range of medical areas. As an illustration of the ambition by the Expert Centre to work with industry, Astra Zeneca has agreed to fund 10 research projects at SciLifeLab with 5–10 million USD per year for 5 years. Several of these projects take advantage of biobanks to promote translational medicine.
- *BARC (Biobanking Analysis Resource Catalogue*): To keep researchers in academia and industry up to date with the rapid development of molecular technologies in the various ‘-omic' fields, the Biobanking Analysis Resource Catalogue, BARC,^29^ has recently been created. BARC provides an overview of both standard analysis technologies and unique advanced molecular technologies provided by ‘-omic' service platforms. It is a freely available web resource, listing expertise and molecular analytic capabilities available at research centres and also tools and resources provided by biotech companies. BARC is managed and developed as part of the Swedish biobank infrastructure, BBMRI.se. Initially focused on facilities and resources in the Nordic region, BARC is now being rapidly expanded to encompass the rest of Europe. BARC is positioned to become an important tool for identification and implementation of new potential expert centres, and to standardize and harmonize services offered.
- *Lifandis AS*: Lifandis AS^30^ is a publicly owned commercial entity set-up to serve as a professional interface between Norwegian biobanks and health registries and industry. The company has expertise within project management, data management, epidemiology/real-world data and biomarkers/diagnostics development, and Lifandis AS actively promotes the use of Norwegian biobanks and health registries in industry-financed projects. Lifandis AS can also provide advanced bioanalytical services through a network of highly competent private and academic partners, and the company plans to establish competence and capacity within biostatistics and bioinformatics. Lifandis AS requires all industry projects to be research focused and approved by regional ethics committees, and the aim is to return all project data/results to the involved bio- and databanks. Currently, Lifandis AS has several ongoing industry- funded projects and a long list of potential new projects under discussion/negotiation.
- *EGC (the Estonian Genome Center)*: EGC^31^ is a university R&D institute managing the Estonian Biobank of 52 000 people and operating as an Expert Centre. EGC is running up-to date genotyping and sequencing facility (CSPro and ISO9001: 2008) providing bioinformatics and statistical support and has performed projects for many customers from EU and outside.

## Figures and Tables

**Table 1:** Revenue forecast 2007–2012 for the global market of biomarkers by segments in US$ millions (within parenthesis in m€ based on the average currency rates of the year in question)

*Market segment*	*2005*	*2006*	*2007*	*2012*	*CAGR (%) 2007–2012*
Biomarker discovery	2.044 (1.645)	2.339 (1.864)	2.677 (1.957)	5.843 (4.546)	16.9
Clinical trials	450 (362)	625 (498)	512 (374)	1.761 (1.370)	23.5
Molecular diagnostics	1.698 (1.367)	1.95 (1.55)	2.3 (1.7)	5.156 (4.011)	17.5
Total	4.192 (3.375)	4.814 (3.837)	5.589 (4.086)	12.76 (9.93)	18

Abbreviations: CAGR, compound annual growth rate.

*Source*: BCC Market Research on biomarkers 2007.^7^

**Table 2:** Revenue forecast 2010–2015 for the global biomarkers market and the genomics segment in US$ millions (within parenthesis in m€ based on the average currency rates of 2010 and 2014)

*Market segment*	*2010*	*2015*	*GAGR (%) 2010–2015*
Biomarkers total	13.5 (10.2)	33.3 (24.9)	19.8
Genomics segment	5.1 (3.9)	16.9 (12.6)	26.9

Abbreviations: CAGR, compound annual growth rate.

*Source*: BCC Market Research on biomarkers 2011.^7^

**Table 3:** Pharmaceutical companies and Biotech firms^a^ having expressed an interest in BBMRI-ERIC and Expert Centres

*AgileBio*	*Geneservice Ltd*	*RNTech*
Alphelys Lab Technologies AstraZeneca	GenVault Corporation	Roche Laboratories
BioKryo GmbH	Genzyme Corp	Sanofi-Adventis
Biomérieux Alliance Biopharmaceutiques	Glaxo-Smith Kline	Semmelweis Inno Center
Biostór Ireland	Imagene	Skinethic
BioStorage Technologies GmbH	Initial R&D Consulting MacoPharma Laboratory	Steelgate SPRL
Ceiso	Merck	TcLand Expression
CyBioFrance	Modul-Bio	Transgene
Euraccine Consulting	Pfizer	Trans-Hit Biomarkers
European Diagnosis Manufacturers	Research Center C Delorme – Air Liquide	VITRO Ltd

a Participants in BBMRI infrastructure, attendees of the Stakeholders's Forum/Meetings or participants of the Expert Centre meeting.

## Notes

Some of the authors are participants in the following Expert Centre examples mentioned: JG, UL – BARC; AM – Estonian Genome Center; CN, UL – SciLifeLab; OVS – Lifandis AS; KZ, CL – Excemet. Of these, Lifandis AS is a publicly owned commercial interface between Norwegian biobanks and health registries and industry. The other entities are public domain activities, receiving public and private project funding.
